# Alleviating Ultrafiltration Membrane Fouling Caused by Effluent Organic Matter Using Pre-Ozonation: A Perspective of EEM and Molecular Weight Distribution

**DOI:** 10.3390/membranes13040452

**Published:** 2023-04-21

**Authors:** Kuo Gao, Hong Yang, Haichen Liu, Bingzhi Dong

**Affiliations:** 1 Shanghai Investigation, Design & Research Institute Co., Ltd., Shanghai 200335, China; 2 School of Environment, Harbin Institute of Technology, Harbin 150090, China; 3 School of Environmental Science and Engineering, Tongji University, Shanghai 200092, China

**Keywords:** effluent organic matter, ultrafiltration, membrane fouling, pre-ozonation

## Abstract

Wastewater reclamation has gradually become an important way to cope with the global water crisis. Ultrafiltration plays an imperative part as a safeguard for the aim but is often limited by membrane fouling. Effluent organic matter (EfOM) has been known to be a major foulant during ultrafiltration. Hence, the primary aim of this study was to investigate the effects of pre-ozonation on the membrane fouling caused by EfOM in secondary wastewater effluents. In addition, the physicochemical property changes of EfOM during pre-ozonation and the subsequent influence on membrane fouling were systemically investigated. The combined fouling model and the morphology of fouled membrane were adopted to scrutinize the fouling alleviation mechanism by pre-ozonation. It was found that membrane fouling by EfOM was dominated by hydraulically reversible fouling. In addition, an obvious fouling reduction was achieved by pre-ozonation with 1.0 mg O_3_/mg DOC. The resistance results showed that the normalized hydraulically reversible resistance was reduced by ~60%. The water quality analysis indicated that ozone degraded high molecular weight organics such as microbial metabolites and aromatic protein and medium molecular weight organics (humic acid-like) into smaller fractions and formed a looser fouling layer on the membrane surface. Furthermore, pre-ozonation made the cake layer foul towards pore blocking, thereby reducing fouling. In addition, there was a little degradation in the pollutant removal performance with pre-ozonation. The DOC removal rate decreased by more than 18%, while UV_254_ decreased by more than 20%.

## 1. Introduction

The treatment and resource utilization of wastewater is an important way to cope with the global water crisis and achieve carbon neutrality [1]. Among the wastewater reclamation processes, ultrafiltration is an important technology with stability, high integration and no phase transition [2,3]. However, the consequent membrane fouling has constrained the further spread of ultrafiltration [4]. Membrane fouling can result in a serious reduction in membrane flux and service life, a sharp increase in energy and cleaning chemicals consumption, and ultimately carbon emissions increase during the process.

Ultrafiltration fouling involves not only the characteristics of the foulants but also the interaction between the foulants and the membrane and the interaction between the foulants, which is much more complicated with the wide range of foulants in water with different physical and chemical properties. Secondary effluent organic matter (EfOM) is considered to be the main source of foulant during advanced wastewater treatment with ultrafiltration [5]. EfOM is a complex organic matrix consisting of a variety of organics with varying molecular weights (<1 kDa~>1000 kDa) [6]. According to the sieving mechanism of low-pressure membranes, large molecular weight (MW) organics will block the membrane pores, while small MW organics can enter inside the membrane pores. Therefore, EfOMs have different potentials for membrane fouling [7,8]. Fan [9] and Kimura [10] et al. have pointed out that large MW organics (biomolecules, etc.) are the key contributors to membrane fouling. However, some researchers [11,12,13,14] have also found that low MW organics exhibited a much higher fouling potential due to their lower MW and higher concentration. The differences or even opposite conclusions of the experimental findings of different researchers are mainly due to the heterogeneous nature of EfOM, and there is an urgent need for an adequate understanding of ultrafiltration fouling caused by EfOM.

Although quite a lot of studies have pointed out that pre-ozonation is an effective measure to mitigate membrane fouling, there is no uniform understanding of the mechanism of mitigation of membrane fouling by pre-ozonation [15,16,17]. It is assumed that pre-ozonation breaks down large MW organics into smaller ones that easily pass through the membrane. Notably, pre-ozonation does not result in complete mineralization of the organic matter, and Karnik et al. [18] showed that the removal of humic acid organics during the pre-ozonation-ultrafiltration process was around 50%, while non-humic acid organics in the effluent increased by around 20%. However, the mitigation of membrane fouling by pre-ozonation was not owed to the change in MW of organics in some studies. Byun et al. [19] pointed out that the mitigation of membrane fouling should be due to the changes in chemical properties of organic matter instead of the changes in molecular weight of organic matter. In the experiment carried out by Kim et al., the contact angle of the fouled membrane surface with pre-ozonation treated Suwannee River water decreased from (40 ± 1.5)° to (30 ± 1.8)° compared to the untreated. Thus, Kim et al. pointed out that pre-ozonation can increase the hydrophilic organics in the feed water, resulting in a fouling resistant layer on the membrane surface [20]. Furthermore, Ting Jiang et al. suggested that pre-ozonation aggravated membrane biofouling at the early stage but alleviated biofouling at the late stage [16]. As mentioned before, the mechanism of membrane fouling alleviation by pre-ozonation needs to be studied more thoroughly and systematically.

In addition, the conditions of the pre-ozonation process also need to be further studied. After a ceramic ultrafiltration fouling experiment by BSA on a lab scale, Song Jia et al. pointed out that ozonation at low dosage slightly mitigates BSA-based membrane fouling, while severe BSA-based membrane fouling was found at high ozone dosage (>4 mg/L) for the BSA aggregates formed through crosslinks [21]. Furthermore, the low MW organics produced during pre-ozonation could easily enter the membrane pores and form serious fouling [8].

In this study, a systematical investigation was carried out to elucidate the membrane fouling caused by EfOM and the fouling alleviation mechanism of pre-ozonation. The fouling behavior of EfOM with pre-ozonation treated during UF was studied through advanced organic matter characterization methods, including high-pressure size exclusion chromatography (HPSEC) and 3-dimensional fluorescence spectroscopy coupled with parallel factor analysis (PARAFAC). Moreover, the combined mechanism (CM) model was used to evaluate the fouling mechanism of EfOM fouling.

## 2. Materials and Methods

### 2.1. Experimental Setup

The experimental laboratory setup adopted dead-end filtration, and its photo is shown in the Supplementary Material (Figure S1). The description of the experimental setup in detail can be found in our previous study [22]. The membrane module was composed of a single polyethersulfone membrane fiber (0.9 mm Multibore^®^, inge, GmbH, Greifenberg, Germany) sealed in a polymethyl methacrylate tube with a mixture of polyurethane and epoxy resin. The modules had an effective membrane surface area of 39.6 cm^2^. The experiments were carried out at a temperature of 25 ± 1 °C, controlled by a water bath thermostat (DKB1915, Jinghong Laboratory Equipment Co., Ltd., Shanghai, China).

Ozone was prepared by a generator using high-purity oxygen as an air source (COM-AD-01, ANSEROS, Tübingen, Germany). The concentration of ozone in water was measured by the indigo trisulfonic acid reagent method (Method 8311, Hash Water Analysis Manual, 5th Edition). The ozone contact reactor is customized with PTFE and has a built-in metal titanium tubular aeration head. The excessive ozone is removed by potassium iodide solution (4%) to prevent leakage into the air. After the raw water is dosed with quantitative ozone and fully reacted (30 min), the remaining ozone is blown off using high-purity nitrogen aeration before the next treatment step.

### 2.2. Ultrafiltration of EfOM Samples

The feed water for ultrafiltration experiments was collected from the secondary effluent samples taken from a wastewater treatment plant in Shanghai, and the conventional water quality parameters of raw water can be found in Table S1. All samples were transported to the laboratory within 24 h, then filtrated by 0.45 µm mixed cellulose ester filter (Xinya Co. Ltd., Shanghai, China) and stored in a refrigerator at 4 °C. The dissolved organic carbon (DOC), ultraviolet absorbance (UV_254_) and PH were measured immediately. To facilitate comparison of the experimental data, the raw water was diluted to 5.0 ± 0.2 mg C/L. The feed water was driven through the membrane by a magnetic gear pump under a constant flux (60 L/(m^2^∙h)). The pressure and flow data were returned every 1 min. To reduce experimental error, the experiment was carried out three times at each ozone dosage. Before every filtration experiment, pristine membrane modules were operated with Milli-Q water for 24 h to remove the organic residuals and wetting agents on the membranes and reached a constant operating pressure.

The experiment procedure was controlled by a programmable logic controller (SIMATIC S7-1200, Siemens, Munich, Germany). The procedures and specific parameters are listed in Table 1.

### 2.3. Analytic Method

The dissolved organic concentrations (DOC) were measured using a total organic carbon (TOC) analyzer (TOV-VCPH, Shimadzu, Kyoto, Japan). Ultraviolet (UV) light absorption at 254 nm was measured by a UV-Vis spectrophotometer (DR5000, HACH, Ames, IA, USA). The molecular weight distribution of EfOM was determined using a high-pressure size exclusion chromatography (HPSEC) method with a UV detector (UVA, Waters 2489, Waters, Milford, MA, USA) and online DOC detector (TOC, Sievers 900 Turbo TOC, GE, Boston, MA, USA). 

Fluorescence excitation–emission matrix spectra (EEM) measurements were carried out with a fluorescence spectrometer (F-7100, Hitachi, Tokyo, Japan). The excitation and emission slits were set to 5.0 nm, and excitation wavelengths were incrementally increased from 200 nm to 450 nm with a step of 10 nm. For each excitation wavelength, the emission wavelengths were detected at a 2.0 nm gap from 275 nm to 575 nm. PARAFAC was used to decompose the fluorescence components in the dataset of 30 EEMs. PARAFAC analysis was conducted using the DOMfluor toolbox [23] in Matlab (version 2016, MathWorks Inc., Natick, MA, USA).

The morphology of the membrane surface and the surface roughness were analyzed using an atomic force microscope (AFM, FM-NanoviewOp-AFM, FSM-Precision Instruments Co., Suzhou, China). Fouled membranes (approximately 1 cm^2^) were stuck to the magnetic stainless steel substrates, and the surface was imaged at a scan size of 5 µm × 5 µm. AFM images were imported to the Gwyddion software to distinguish the height of samples by the color scale and then to calculate the surface roughness.

### 2.4. Membrane Fouling Analysis

The membrane fouling behavior can be evaluated by the resistance-in-series model shown below:

(1) $$R_{t}=\frac{∆P}{\mu J}=R_{m}+R_{ir}+R_{r}$$

(2) $$R_{r}=\frac{∆P_{n_{t}}-∆P_{{\left(n+1\right)}_{0}}}{\mu J}$$

(3) $$R_{ir}=\frac{∆P_{{\left(n+1\right)}_{0}}-∆P_{n_{0}}}{\mu J}$$

where *R_t_*, *R_m_*, *R_r_* and *R_ir_* are the total resistance during the ultrafiltration (m^−1^), the resistance of intrinsic membrane resistance (m^−1^), hydraulically reversible resistance (m^−1^) and hydraulically irreversible resistance (m^−1^), respectively. *μ* is the dynamic viscosity of the synthetic feed water (Pa∙s), ∆P the trans-membrane pressure (kPa) and the subscript *n*, *t*, *0* are the filtration cycle number, end of every filtration cycle and initial stage of every filtration cycle. Notably, the average pressure value over 5 min is adopted as the calculated pressure to avoid the sensor data deviation.

Additionally, *J* is the permeate flux during the ultrafiltration process (m/s), which can be calculated as:

(4) $$J=\frac{Q}{A}$$

where Q is the permeate flow rate (m^3^/s) recorded by the flow transmitter (LSF38, OVAL, Japan) and *A* is the effective membrane surface area (m^2^).

## 3. Results and Discussion

### 3.1. Fouling Behavior during Ultrafiltration

**Figure 1:**
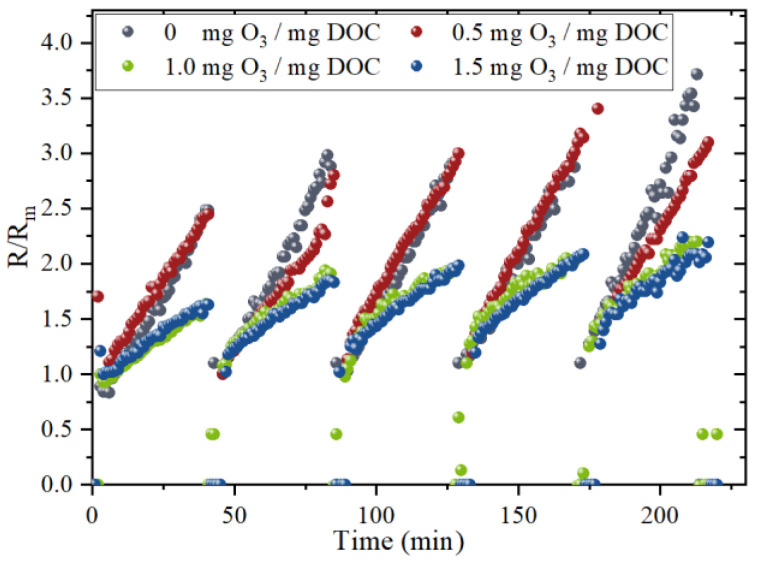
The normalized resistance variation during ultrafiltration with different pre-ozonation dosages.

The normalized resistance variation during the experiment with different ozone dosages is shown in Figure 1. The normalized total fouling resistance at the end of the 5th cycle of raw water with 0 mg O_3_/mg DOC treated reaches 3.71, which is similar to the final resistance (3.10) of raw water with 0.5 mg O_3_/mg DOC treated. Since ozone in low concentration also degrades organic matter effectively due to its strong oxidative characteristics, it is usually hypothesized that a low level of ozone is preferred to oxidize little of organics to inorganics, which shows little alleviation rather than more oxidizing high MW organics to low MW organics [24]. However, the changes in molecular weight distribution of EfOM after pre-ozonation, which was illustrated in Section 3.2, were contrary to the former hypothesis. The concentration of organic matter with low MW reaches a relative peak, while organic matter with high MW showed significant removal. This suggests that the poor alleviation effect should be due to a synergistic effect of low MW organic matter, which results in a deterioration of membrane fouling.

Notably, the normalized resistance at the end of the 5th cycle of raw water with 1.0 and 1.5 mg O_3_/mg DOC treated are 2.08 and 2.05, respectively. With the increase of ozone dosage, the high MW organic matter is further oxidized and decomposed into low MW organic matter, while some organic matter is directly mineralized (as shown in Section 3.2). However, the alleviation by pre-ozonation with 1.5 mg O_3_/mg DOC shows no significant improvement compared with the pre-ozonation with 1.0 mg O_3_/mg DOC (Figure 1). This is mainly because there is no further removal of macromolecular organics (as shown in Section 3.2), which is also proved in Tang’s study [25].

**Figure 2:**
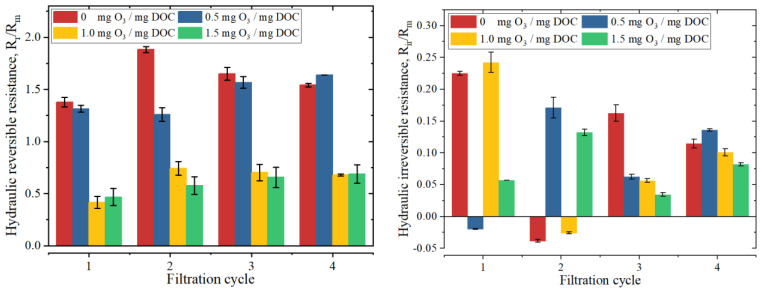
The normalized resistance variation during ultrafiltration with different pre-ozonation dosages (R_r_/R_m_, normalized hydraulically reversible resistance; R_ir_/R_m_, normalized hydraulically irreversible resistance).

Figure 2 illustrates the changes in hydraulically reversible resistance and hydraulically irreversible resistance of the ultrafiltration membrane with different ozone dosages. With a dosage of 0 mg O_3_/mg DOC, the hydraulically reversible resistance (R_r_/R_m_) ranges from 1.37 to 1.88. R_r_/R_m_ decreases significantly to 0.41~0.74 with 1.0 and 1.5 mg O_3_/mg DOC, which is mainly for the removal of high MW organic matter and the mineralization of organic matter. Although there is a great fluctuation in the hydraulically irreversible resistance (R_ir_/R_m_), R_ir_/R_m_ increases first and then decreases as the ozone dosage increases.

### 3.2. Changes in the Properties of EfOM

**Figure 3:**
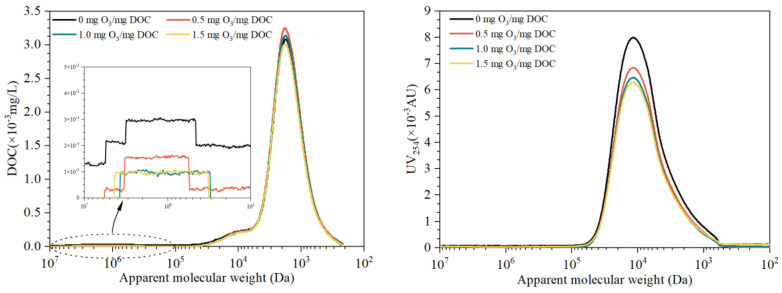
Changes in molecular weight distribution of EfOM after pre-ozonation: DOC response in left, UV_254_ response in right.

The apparent molecular weight distribution (AMWD) in EfOM samples with the same DOC concentration (5.0 ± 0.2 mg C/L) after pre-ozonation is shown in Figure 3. AMWD in different EfOM samples can be divided into three regions [26,27], i.e., low molecular region III (500–7000 Da), medium molecular region II (4000–40,000 Da) and high molecular region I (600,000–2,000,000 Da). Of note, organic matters in region I show a rare UV_254_ response, indicating the low aromatic structure of high MW organic matters; organics in region II has the strongest UV_254_ response along with the moderate DOC response, and organic matters in region III account for most DOC response with lower UV_254_ response. With the ozone dosage increase, the DOC response in region I decreases significantly. While the UV response decreases significantly in region II, no clear DOC response reduction is observed in region II. This indicates that ozone has a strong ability to destroy the unsaturated structure of organic matter and unsaturated organic matter with a strong UV response [28]. The UV response of organic matter in region III shows a dramatic decrease with pre-ozonation, but the DOC response is higher than the raw water with relatively low ozone dosages (0.5 and 1.0 mg O_3_/mg DOC). The increase is mainly due to the low MW organic matter produced by the decomposition of high MW organic matter.

**Figure 4:**
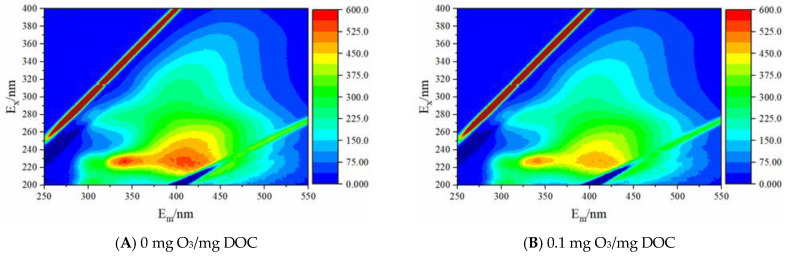

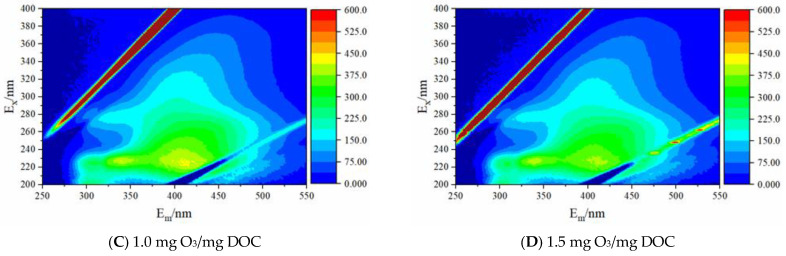
EEMs of EfOMs after pre-ozonation with different ozone dosage.

**Figure 5:**
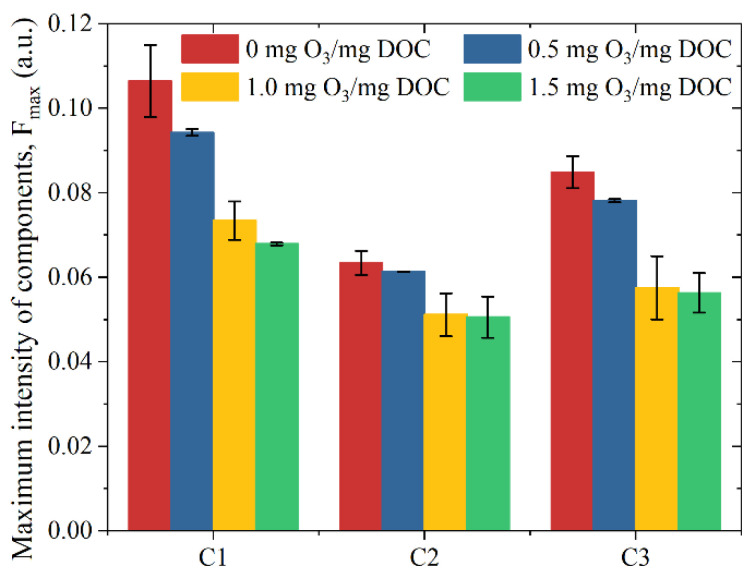
F_max_ of fluorenscence components of EfOM after pre-ozonation.

The EEMs of the EfOMs treated with different ozone dosages are shown in Figure 4. The fluorescence intensity of protein-like and fulvic-like in the EfOMs is significantly decreased with the increase of ozone dosage during the pre-ozonation, which further demonstrates the ability of ozone to destroy unsaturated structures (aromatic structures). The changes in the maximum intensity of each fluorescence component obtained by the parallel factor method decomposition are shown in Figure 5.

The intensity of the humic acid-like component (C1) and microbial metabolites, and aromatic protein component (C3) decreases significantly with the increase of ozone dosage, while the tryptophan-like protein component (C2) shows little reduction, which is consistent with the results of Yu Huarong et al. [29]. That is mainly because ozone is selective for organic matter and can easily oxidize organic substances with C=C, benzene ring structure.

### 3.3. Pollutant Removal Performance

**Figure 6:**
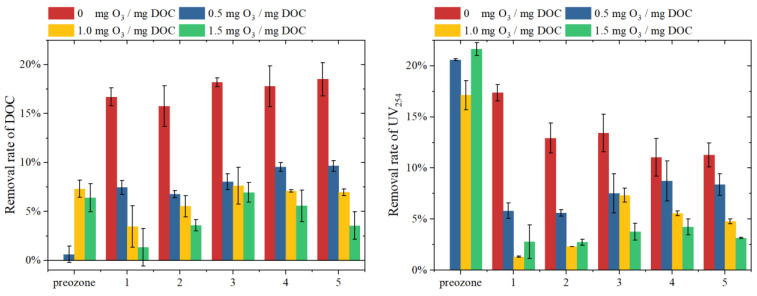
Pollutant removal of pre-ozonation and ultrafiltration under different ozone dosage.

The variation of DOC and UV_254_ removal after the pre-ozonation and subsequent ultrafiltration are shown in Figure 6. During the pre-ozonation, the removal of DOC gradually increased with the increase of ozone dosage. Notably, there is no significant removal of DOC with the ozone dosage of 0.5 mg O_3_/mg DOC. This is mainly because that ozone tends to oxidize high MW organic matter to medium or low MW organic matter than mineralization directly at low ozone concentration (Figure 3). There is no significant difference in DOC removal during pre-ozonation with 1.0 and 1.5 mg O_3_/mg DOC, which is about 6~7%. The DOC removal rate of the EfOM ultrafiltration is about 15~20% with 0 mg O_3_/mg DOC treated. In addition, the subsequent DOC removals during the EfOM ultrafiltration slowly decrease with the increasing ozone dosage, which should be owed to the fact that the decomposed low MW organic matter can easily pass through the membrane. The removal rate of UV_254_ in the pre-ozonation stage is about 17.1~21.6%, which is much higher than the DOC removal rate (~7%).

**Figure 7:**
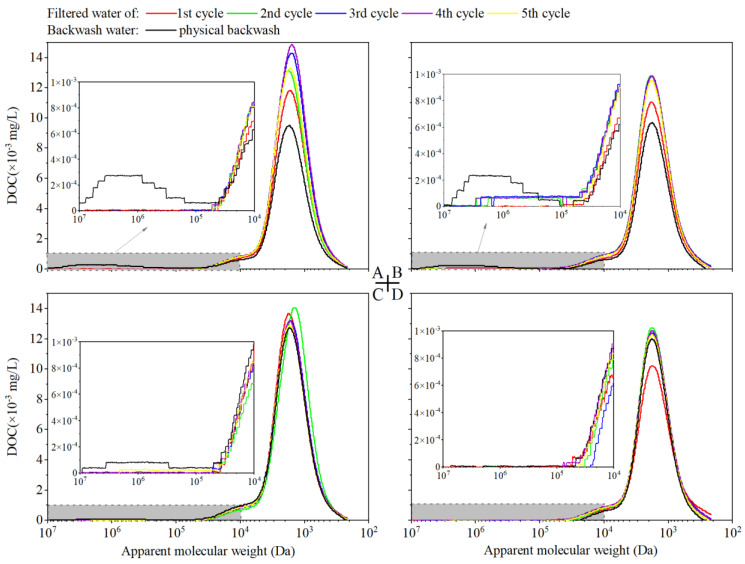
Molecular weight distribution of NOM in filtrated water and physical cleaning water: (**A**) 0 mg O_3_/mg DOC; (**B**) 0.5 mg O_3_/mg DOC; (**C**) 1.0 mg O_3_/mg DOC; (**D**) 1.5 mg O_3_/mg DOC.

The molecular weight distributions of organic matter in the filtered water and physical cleaning solution in each cycle with different ozone dosages are shown in Figure 7. The DOC response of low MW organic matter in filtered water at the first cycle is quite low for the adsorption effect of the membrane. While the relatively low DOC response also can be found in the fifth cycle, which is mainly for the synergistic effect of high MW and low MW resulting in the increased removal of low MW organic matter. Due to the oxidation of high MW organic matters by pre-ozonation and the serious irreversible membrane fouling, the DOC responses of high MW organic matters in the physical cleaning solution decrease.

### 3.4. Fouling Mechanism Analysis

**Figure 8:**
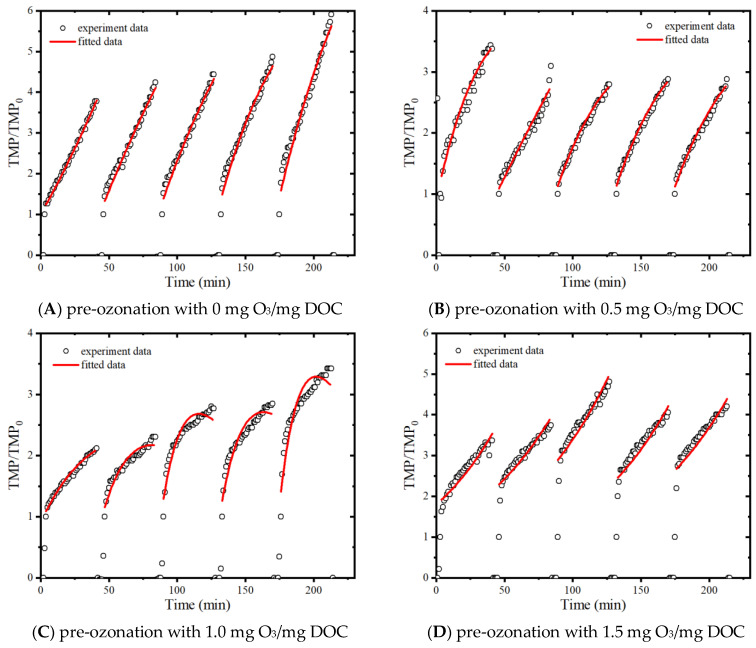
Fouling model of ultrafiltration with EfOM treated with different ozone dosages.

The combined mechanism models, whose detailed description can be found in our previous studies [22,30], are used to analyze the fouling data, and the fit result is presented in Figure 8 and Table 1. Since the terms $K_{s}$, $K_{i}$ and $K_{c}J_{0}$ have similar units (m^−1^), the contributions of individual fouling mechanisms could be evaluated by comparison of their obtained fitted parameter values [31]. Obviously, the dominant fouling mechanism has changed from cake layer fouling to standard pore-blocking fouling with the ozone dosage increases.

During pre-ozonation with 0 mg O_3_/mg DOC, the fouling mechanism is cake layer fouling + intermediate pore blocking, and the cake layer fouling is dominant ($K_{i}/(K_{c}*J_{0}$) = 0.02~0.06). When the ozone dosage increases to 0.5 mg O_3_/mg DOC, there is no significant change in the fouling mechanism, and the percentage of cake layer fouling decreases slightly ($K_{i}/(K_{c}*J_{0}$) = 0.02~0.10). Combined with the results of membrane surface morphology (Table 2), it is suggested that the structure of the cake layer becomes denser, and the thickness of the cake layer decreases due to the oxidation and decomposition of high MW organic matter into low MW organic matter by ozone. Therefore, the fouling resistance of the membrane does not show much variation under the lower ozone dosage. The percentage of cake layer fouling decreases significantly ($K_{i}/(K_{c}*J_{0}$) = 0.12~0.51) when the ozone dosage increases to 1.0 mg O_3_/mg DOC. More high MW organic matter is oxidized by ozone, and some low MW organic matter is removed by direct mineralization. The percentage of intermediate pore-blocking fouling is elevated, and the roughness and thickness of the fouled membrane surface also increase more significantly, indicating that the structure of the fouling layer becomes sparser while the corresponding total fouling resistance also decreases significantly (Figure 2). When the ozone dosage further increases to 1.5 mg O_3_/mg DOC, more high MW organic matters are oxidized and decomposed (Figure 3), and the fouling mechanism changes from cake-intermediate fouling to standard-intermediate fouling, and the standard pore blocking is dominant. At this time, the thickness of the fouling layer decreases significantly (from 500.0 nm to 337.4 nm, as shown in Table 2), and the total fouling resistance also decreases to a certain extent (Figure 2). In this experiment, the fouling mechanism changes to standard-intermediate fouling under the effect of higher ozone dosage, while the fouling mechanism changes to cake-standard fouling in the experiment of Song Jia et al. [21]. This is mainly because the EfOM used in this study is mainly composed of medium and low MW organic matter, while the model foulant used in the experiment of Song Jia et al. is BSA macromolecules.

### 3.5. Analysis of Foulant Layer Morphology

**Figure 9:**
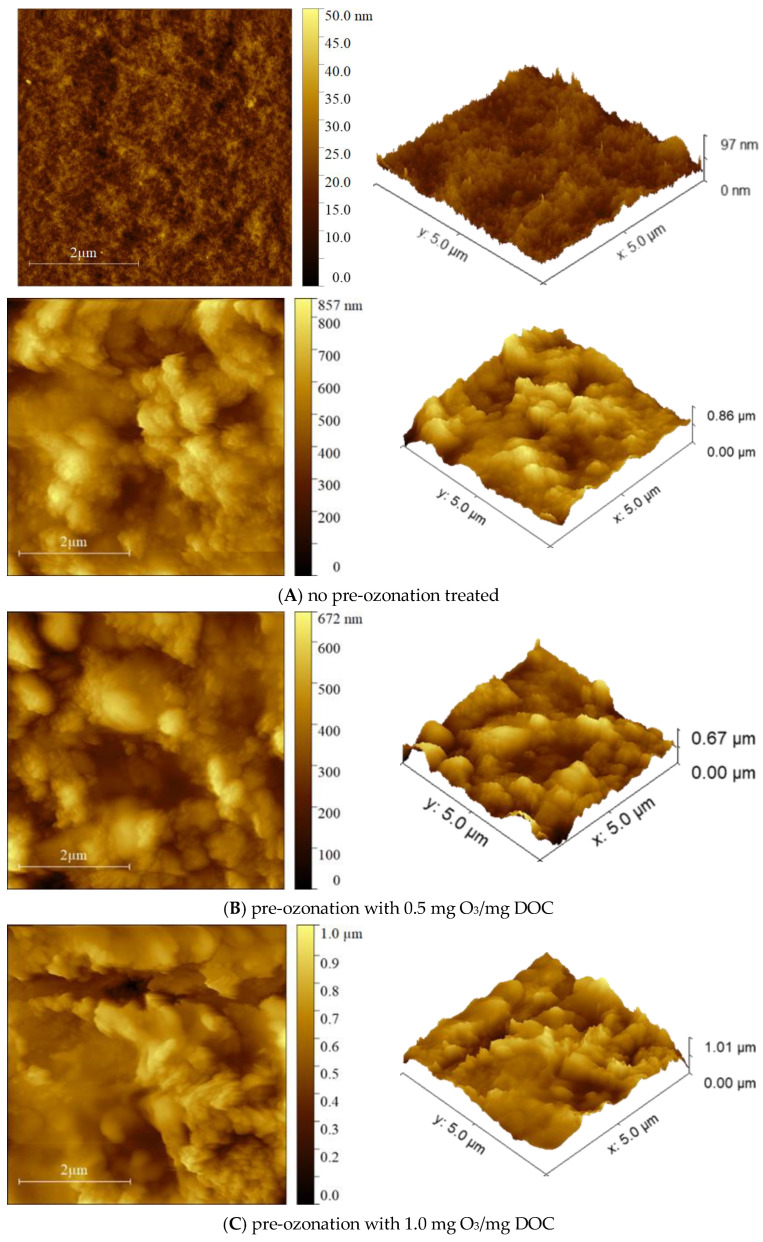

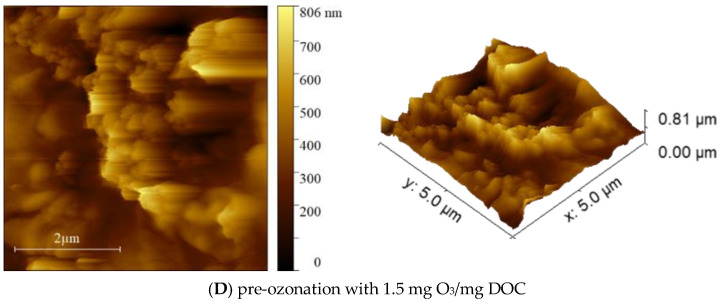
AFM morphology of fouled membranes caused by EfOM with different pre-ozonation dosages.

Figure 9 shows the surface morphology of the membrane fouled by EfOM treated with different ozone dosages. Compared with the pristine membrane, the fouled membrane has an obvious fouling layer formation, and the structure of the fouling layer differs significantly under different ozone dosages. As shown in Table 3, the roughness of the fouled membrane is relatively low, and the average thickness of the fouling layer is high when the ozone dose is 0 mg O_3_/mg DOC.

When the ozone dose is 0.5 mg O_3_/mg DOC, low MW organic matters increase in the feed water, the average thickness of the fouling layer decreases significantly, and the roughness of the fouling layer also decreases significantly. This indicates that the low MW organic matters make the fouling layer on the membrane surface denser, which is also reflected in the change of fouling resistance during filtration (Figure 2). With 1.0 mg O_3_/mg DOC treated, the high MW organic matter is further oxidized and decomposed, the low MW organic matter is partially mineralized by ozone, and the structure of the fouling layer is similar to the low MW organic matter alone filtration. In addition, the surface roughness of the fouling layer becomes larger, and the average thickness becomes higher. When the dosage reaches 1.5 mg O_3_/mg DOC, the low MW organic matter is further mineralized, and the roughness of the fouling layer on the membrane surface does not change much, but the average thickness of the fouling layer decreases.

## 4. Conclusions

EfOMs treated with different ozone dosages are used in the ultrafiltration experiment. The major conclusions are summarized as follows:

1. The apparent molecular weight distribution of EfOMs shows that pre-ozonation is effective in removing high MW organic matters with 0~1.5 mg O_3_/mg DOC dosage, and the removal of low MW organic matters is limited.
2. The fluorescence intensity of aromatic protein-like and fulvic-like in the EfOM is significantly weakened with the increase of ozone dosage. This further demonstrates the ability of ozone to destroy unsaturated structures (aromatic structures). The intensity of the humic-like component (C1) and microbial metabolites and aromatic protein component (C3) decreases significantly with the increase of ozone dosage, while the tryptophan-like protein component (C2) decreases to a lesser extent.
3. The removal rate of organic matter during the subsequent ultrafiltration is influenced by the pre-ozonation, which gradually decreases with the increase of ozone dosage. It indicates that pre-ozonation may have a negative impact on the effluent quality of ultrafiltration.
4. With a relatively low dosage (0.5 mg O_3_/mg DOC), the fouling layer is denser but lower in thickness, which has no obvious effect on the membrane fouling resistance. When the ozone dosage increases to 1.0 or 1.5 mg O_3_/mg DOC, the structure of the fouling layer is looser, and the normalized final fouling resistance is 2.05 or 2.08, respectively. There is obviously alleviation in the ultrafiltration fouling.

## Figures and Tables

**Table 1:** Parameters of ultrafiltration system operating conditions during a filtration cycle.

Procedure	Flow (mL/min)	Duration (min)
Discharge	8.0	1
Filtration ^1^	4.0	40
Forward wash ^2^	8.0	1
Backwash ^2^	8.0	2

^1^ The constant flux in this study was 60 L/(m^2^·h). ^2^ The flow of forward wash and backwash were determined by manufacturer’s recommendation.

**Table 2:** Parameters of the fouling mode of ultrafiltration with raw water treated by pre-ozonation.

**Dosage of Ozone** **during Pre-Ozonation**	**Fouling Model**	**Filtration** **Cycle**	***K_i_***	***K_c_* × *J*_0_**	$\left|\frac{K_{i}}{K_{c}\times J_{0}}\right|$	**R^2^**
0 mg O_3_/mg DOC	Cake-intermediate	1	94.77	2386.27	0.04	0.9954
		2	−71.85	3458.13	0.02	0.9813
		3	−144.03	3963.60	0.04	0.9842
		4	−241.49	4012.56	0.06	0.9780
		5	−191.30	4305.41	0.04	0.9710
0.5 mg O_3_/mg DOC	Cake-intermediate	1	−354.25	3671.82	0.10	0.9628
		2	−33.80	2231.41	0.02	0.9511
		3	−272.83	3460.10	0.08	0.9871
		4	−265.15	3498.78	0.08	0.9885
		5	−242.30	2993.28	0.08	0.9800
1.0 mg O_3_/mg DOC	Cake-intermediate	1	−316.63	2361.97	0.13	0.9818
		2	−617.37	4235.04	0.15	0.900
		3	−871.31	1711.72	0.51	0.8822
		4	−755.22	5548.64	0.14	0.8750
		5	−910.48	7602.57	0.12	0.8265
**Dosage of Ozone** **during Pre-Ozonation**	**Fouling Model**	**Filtration** **Cycle**	***K_i_***	***K_s_***	$\frac{K_{i}}{K_{s}}$	**R^2^**
1.5 mg O_3_/mg DOC	Standard-intermediate	1	−686.68	−1007.49	0.68	0.8670
		2	−589.60	−789.26	0.75	0.8084
		3	−598.29	−713.24	0.84	0.7736
		4	−605.52	−783.35	0.77	0.8013
		5	−555.22	−687.64	0.81	0.7581

**Table 3:** Morphology parameters of fouled membrane.

Dosage of Ozone during Pre-Ozonation	Mean Roughness *S_a_* (nm)	Average Thickness of Foulant Layer *h* (nm)
pristine membrane	8.3	53.2
0 mg O_3_/mg DOC	87.6	500.0
0.5 mg O_3_/mg DOC	75.6	344.7
1.0 mg O_3_/mg DOC	93.2	561.0
1.5 mg O_3_/mg DOC	90.5	337.4

## Data Availability

The data that support the findings of this study are available from the corresponding author upon reasonable request.

## Supplementary Materials

The following supporting information can be downloaded at: https://www.mdpi.com/article/10.3390/membranes13040452/s1, Figure S1: Photos of laboratory experimental setup and homemade membrane modules; Figure S2: Contour plots of the three components identified from EEMs dataset; Table S1: Analysis of conventional water quality parameters; Table S2: Description and wavelength positions of PARAFAC components, and their comparisons with previously identified components. References [32,33,34,35,36] are cited in the Supplementary Materials.
