# Supplementary material for: Alleviating Ultrafiltration Membrane Fouling Caused by Effluent Organic Matter Using Pre-Ozonation: A Perspective of EEM and Molecular Weight Distribution
Source: Membranes (Basel). 2023 Apr 21;13(4):452. doi: 10.3390/membranes13040452 (PMC10141980; doi:10.3390/membranes13040452)
Supplement: Supplementary file 1 [file membranes-13-00452-s001.zip › membranes-2330498-supplementary.pdf]

Supplementary Material

Alleviating Ultrafiltration Membrane Fouling Caused by Effluent Organic Matter Using Pre-Ozonation: A Perspective of EEM and Molecular Weight Distribution

The photos of homemade laboratory experimental setup and membrane module were shown in Fig. S1.

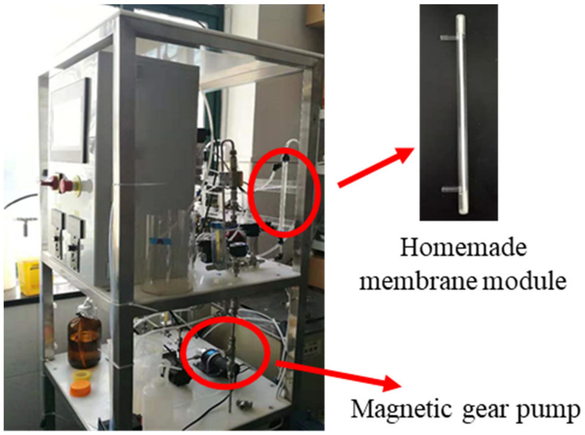

Figure S1. Photos of laboratory experimental setup and homemade membrane modules.

Table S1. Analysis of conventional water quality parameters.

Parameters	pH	DOC (mg C/L)	COD (mg O_2/ L)	UV_{254} (cm^{-1})
Maximum	7.35	6.85	38.7	0.168
Minimum	6.76	4.92	31.2	0.123
Mean	7.02	5.42	35.2	0.145

The identified components of PAFARAC were shown in Fig. S2. And the previous studies on description and wavelength positions of PARAFAC components can be found in Table S2.

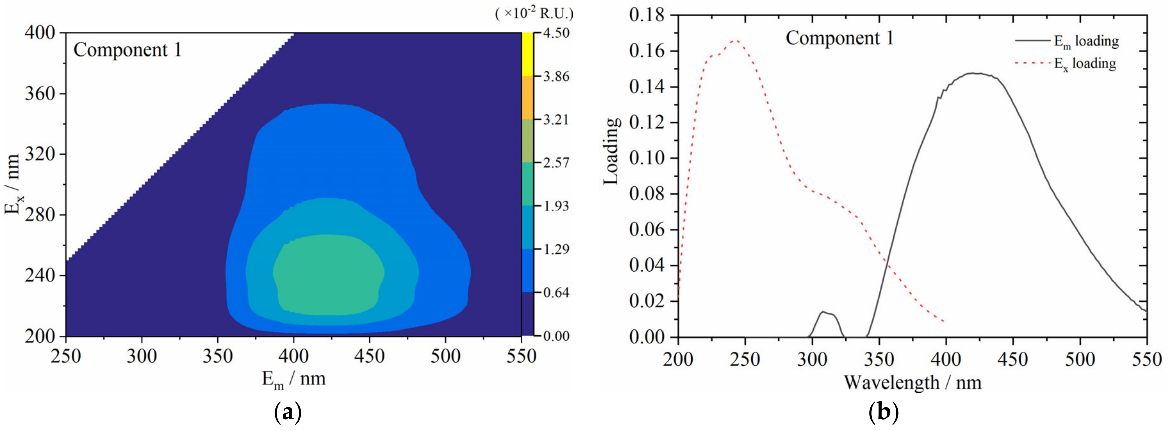

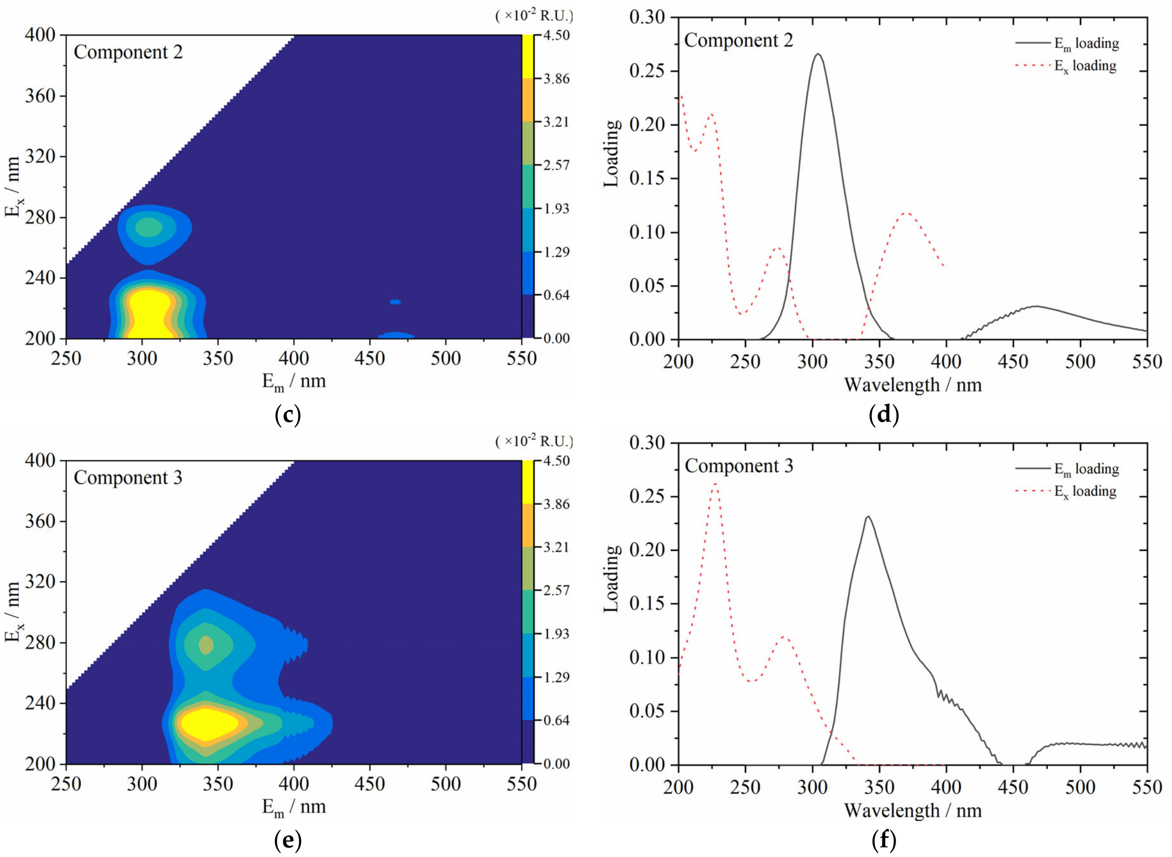

Figure S2. Contour plots of the three components identified from EEMs dataset. The right hand side plots show the split-half validations of excitation (red) and emission (black) loadings.

Table S2. Description and wavelength positions of PARAFAC components, and their comparisons with previously identified components.

**This Study**		**Previous Studies**		
**Fluorescent component**	**Position (E_x/E_m)**	**Position (E_x/E_m)**	**Description**	**Reference**
Component 1	240/430	<250/400	Microbial humic-like fluorescence	[1]
		<240-260/374-450	Humic-like compounds	[2]
Component 2	235, 275/300	270/304	Protein, tyrosine-like	[1]
		275/340	tryptophan-like	[3]
Component 3	225, 280/340	300/342	Aromatic protein	[4]
		280/330	Protein-like fluorescence, associated with microbial activity and biological productivity	[5]

References

1. Murphy, K.R.; Hambly, A.; Singh, S.; Henderson, R.K.; Baker, A.; Stuetz, R.; Khan, S.J. Organic Matter Fluorescence in Municipal Water Recycling Schemes: Toward a Unified PARAFAC Model. *Environ. Sci. Technol.* **2011**, *45*, 2909–2916, https://doi.org/10.1021/es103015e.
2. Ishii, S.K.L.; Boyer, T.H. Behavior of Reoccurring PARAFAC Components in Fluorescent Dissolved Organic Matter in Natural and Engineered Systems: A Critical Review. *Environ. Sci. Technol.* **2012**, *46*, 2006–2017, https://doi.org/10.1021/es2043504.
3. A Stedmon, C.; Markager, S.; Bro, R. Tracing dissolved organic matter in aquatic environments using a new approach to fluorescence spectroscopy. *Mar. Chem.* **2003**, *82*, 239–254, https://doi.org/10.1016/s0304-4203(03)00072-0.

4. Yamashita, Y.; Scinto, L.J.; Maie, N.; Jaffé, R. Dissolved Organic Matter Characteristics Across a Subtropical Wetland's Landscape: Application of Optical Properties in the Assessment of Environmental Dynamics. *Ecosystems* **2010**, *13*, 1006–1019, https://doi.org/10.1007/s10021-010-9370-1.
5. Seredyńska-Sobecka, B.; Stedmon, C.A.; Boe-Hansen, R.; Waul, C.K.; Arvin, E. Monitoring organic loading to swimming pools by fluorescence excitation–emission matrix with parallel factor analysis (PARAFAC). *Water Res.* **2011**, *45*, 2306–2314, https://doi.org/10.1016/j.watres.2011.01.010.
